# A Comparison Between Italian Division I and College American Football Players in the NFL Combine Test Battery

**DOI:** 10.3390/jfmk10010008

**Published:** 2024-12-27

**Authors:** Federico Nigro, Sandro Bartolomei, Alessio D’Amico, Simone Ciacci, Rocco Di Michele, Vittorio Coloretti, Matteo Cortesi

**Affiliations:** Department for Life Quality Studies, University of Bologna, 40126 Bologna, Italy; federico.nigro2@unibo.it (F.N.); alessiodamico13@gmail.com (A.D.); simone.ciacci@unibo.it (S.C.); rocco.dimichele@unibo.it (R.D.M.); vittorio.coloretti2@unibo.it (V.C.); m.cortesi@unibo.it (M.C.)

**Keywords:** player position, stature, weight, normalization, network analysis

## Abstract

**Objectives**: The purpose of the present study was to evaluate the level of physical capacities of Italian American Football (AF) players and compare their performances with published data of American college players. A secondary aim was to assess whether the performance of Italian players in the NFL Combine tests has improved over time compared to previously tested players of similar competitive level. A total of 41 Italian AF players (age 28.1 ± 4.7 y, stature 181.1 ± 5.9 cm, body mass 98.3 ± 17.8 kg) competing in the 2020/2021 Division I Championship, participated in this study and performed the NFL Combine test battery. **Methods**: The NFL Combine test battery includes the 40-yard dash, the 20-yard shuttle, the 3-cone drill tests, the broad jump test, the vertical jump test, and the maximum number of repetitions at bench press with a 100 kg load. Players were divided into three groups based on their playing position: skill players (SP = 14), big skill players (BSP = 9), or linemen (LM = 13). In addition, players’ performance scores were normalized to their stature and body weight. **Results**: Italian players showed lower performances in all the six tests compared to American college players. Significant differences were observed between player positions. Normalized performances were significantly lower in Italian compared to American players. **Conclusions**: Despite an improving trend in the NFL Combine tests being registered in Italian AF players, a relevant gap still exists compared to their US counterparts.

## 1. Introduction

American Football (AF) is one of the most popular sports in the United States (US). The game is characterized by the extraordinary speed and power produced by players during accelerations and changes of direction [1]. Herculean efforts and intense collisions are some of the elements that contribute to the sport’s widespread appeal [2]. Thanks to the outstanding physical skills of the players, remarkable efforts and unique performances can be appreciated in short-lasting actions [3,4]. Although AF has been a dominant sport in the US for decades, its popularity has recently grown in several European countries. For example, interest in this sport recently increased in Italy after the Italian national team won the European Championship in 2021. However, most of the research in this area has been conducted on US players, with only a few studies on European teams [3,5,6,7,8,9,10,11,12].

A quantitative approach to the selection of athletic talents involves the use of a specific battery of physical tests, also known as the NFL Combine. This assessments are recognized as a reliable and valid tool for assessing athletic performance [13]. Specifically, the protocol of the NFL Combine includes the 40-yard dash, 225 lb bench press repetitions to exhaustion, vertical jump, broad jump, pro agility shuttle test, and the 3-cone drill [14]. Players’ performances assessed with NFL Combine tests are published online and accessible for analysis. These data are typically interpreted considering the level of the players [9,14,15], their playing position [12] and age [7]. Furthermore, optimal combinations of scores in the various tests were developed for each playing position [5,13,16].

Extensive scientific literature has analyzed the results of the NFL Combine. Cook et al. examined the relationship between the NFL Combine results and game performance over a five-year period [6]. The average number of games played by athletes in the first two years of their careers was found to be higher for those who performed better at the Combine. Sierer and colleagues analyzed year-to-year differences in Combine performance [14], as did Fitzgerald et al. over a fifteen-year period [8]. NFL Combine results were also used to predict players’ match performance [11,13,16,17]. For example, scouting NFL Combine results are predictive of future performance for specific playing positions, such as running backs and wide receivers [10,18]. Sierer et al. identified performance differences between drafted and non-drafted players [14]. To ensure an accurate evaluation of a player’s performance, it is important to properly standardize NFL Combine test results appropriately. Some researchers suggest normalizing the results by considering multiple specific characteristics of each player, and not only the single test performance [6,16]. A common criterion for comparing players with different characteristics is their playing position. In addition, normalizations based on anthropometric characteristics such as stature and/or weight, are frequently employed. Given these premises, it was deemed appropriate to compare the performance of Italian Division I players to that of US college players rather than senior players.

The primary aim of this study was to compare the performance levels of Italian AF players with those of US college players. Specifically, the purpose was to compare the performances normalized for players’ position, body mass, and stature. A secondary aim was to evaluate whether the performance of Italian Division I AF players has improved in recent years. This was achieved by comparing the current data with data from another Italian Division I team of a similar competitive level that participated in the Italian Championship in 2014. It was hypothesized that Italian players would underperform compared to their US peers in all the combined tests, even after the normalization process was applied. A second hypothesis was that Italian Division I players in this study performed better than their counterparts who were tested in 2014.

## 2. Materials and Methods

### 2.1. Experimental Approach to the Problem

A total of 41 players competing in the 2020/2021 Italian Division I AF Championship participated in the present study. All tests were carried out at the end of the pre-season phase, before the beginning of the national championship, during the last training session of the week [19]. These data were compared with those of US college players. In particular, the data in this study were obtained from the website pro-football-reference.com (accessed on 25 November 2021), which was designed to systematically collect the official results of NFL Combine tests [10,20,21,22]. Given the distinctive physical attributes of college players, their performance at the NFL Combine was the most pertinent for comparison with that of Italian players [23,24]. Finally, the players’ test scores of the present study were compared with data previously collected from Italian Division I players, available from the literature [15].

### 2.2. Participants

The sample size was determined a priori using G*Power software (Version 3.1.9.7). A two-tailed *t*-test for independent samples was selected to evaluate differences in performance metrics between two groups. The effect size (Cohen’s d) was set at 2.0, based on the minimum effect size reported in a previous study [15]. The significance level (α) was set at 0.05, and the statistical power (1-β) was fixed at 0.80 to ensure adequate sensitivity. The allocation ratio between groups was set to 1:1. Based on these parameters, the required total sample size was calculated to be 36. A total of 41 Italian Division I AF players (age 28.1 ± 4.7 y, stature 181.1 ± 5.9 cm, body mass 98.3 ± 17.8 kg) were involved in the present study. All tests were performed with the supervision of the investigators and of two experienced strength and conditioning coaches. Inclusion criteria required players to be between the ages of 18 and 35 years, and to have a minimum of 2 years of NFL Combine testing experience. Participants were asked to abstain from alcohol, caffeine, and resistance training for at least 24 h before the tests. Exclusion criteria included severe injuries that occurred in the year before the study. Finally, five players were excluded from the analysis. According to Sierer et al. [14,15], playing position was categorized as skill player (SP) (*n* = 14), big skill player (BSP) (*n* = 9) or lineman (LM) (*n* = 13). SP players included wide receivers, cornerbacks, free safeties, strong safeties, and running backs; BSP included fullbacks, linebackers, tight ends, and defensive ends; and LM included centers, offensive guards, offensive tackles, and defensive tackles. Quarterbacks, kickers, and punters were tested but not included in the analyses in agreement with previous authors [14,15].

### 2.3. Procedures

Field tests were performed on an artificial turf pitch. All players performed a standardized 20 min warm-up consisting of five minutes of aerobic jogging, 10 body weight squats, 10 body weight walking lunges, 10 dynamic walking hamstring stretches, 10 dynamic walking quadriceps stretches and 10 body weight push-ups [25,26]. The warm-up was supervised by a qualified strength and conditioning coach [4]. All players performed the warm-up without helmets or shoulder pads, and were allowed to drink water ad libitum during breaks. Warm-up included dynamic movements and activation exercises for all joints and muscles. The time taken to perform the 40-yard dash, 20-yard shuttle and 3-cone drill tests was measured using photocells (FitLight Corp, Aurora, ON, Canada). The jump length in the broad jump test was measured using a tape measure. Vertical jump was performed with a countermovement with free arms, and jump height was measured using a contact mat (Chronojump Bosco System, Barcelona, Spain). The maximum number of repetitions at bench press with a 100 kg load was determined as previously described by Sierer and colleagues [14]. With the exception of the maximum bench press test, the best of the two attempts was recorded for each test. To compare the NFL Combine test battery results between the present sample of AF players and data available from the literature, the data reported by Vitale et al. [15], collected on Division I Italian AF players in 2014, were used. Results of American college players were obtained online, as previously described by Sierer et al. [14]. In addition, since the physical size of players may affect the absolute results of fitness tests, we normalized the players by both playing position and two additional criteria (stature and body weight). Among all US college players available in the data set, those with similar stature and weight as the Italian players were selected for the present analysis. The criterion adopted by the researchers was the smallest possible numerical gap between US college players and Italian players. The two investigators involved in the player selection process made identical decisions. Finally, 36 drafted and 36 non-drafted American players a stature of ±10 cm and a weight of ±5 kg compared to the sample Italian players, were included into the analysis.

### 2.4. Statistical Analysis

For all examined variables, the assumption of distribution normality was checked with Shapiro–Wilk tests. The effects of group and playing position, and their interaction, was assessed with 2-way analysis of variance (ANOVA). If a significant difference was detected in the main analysis, Tukey’s post hoc test was conducted. When the assumption of normality was not satisfied, an Aligned Rank Transformation was used before performing subsequent analysis. The results of Italian players were compared with the results of their counterparts in 2014, using Welch’s unpaired t-test. The analyses were performed with the software R, version 4.3.1 (The R Foundation for Statistical Computing, Vienna, Austria), except for network analysis that was performed using JASP (version 0.16) setting EBICglasso as estimator (Graphical Least Absolute Shrinkage and Selection Operator with Bayes’ extended information criterion), γ = 0.5 and in case of missing values we adopt pairwise exclusion criteria. The level of statistical significance was set at *p* ≤ 0.05 for all the analyses.

## 3. Results

The NFL Combine results of the Division I Italian players and of the US college players are reported in Table 1 as mean and standard deviations. The data of 36 Italian players and their respective drafted and undrafted counterparts, normalized by position, stature and body weight, are presented.

**Figure 1:**
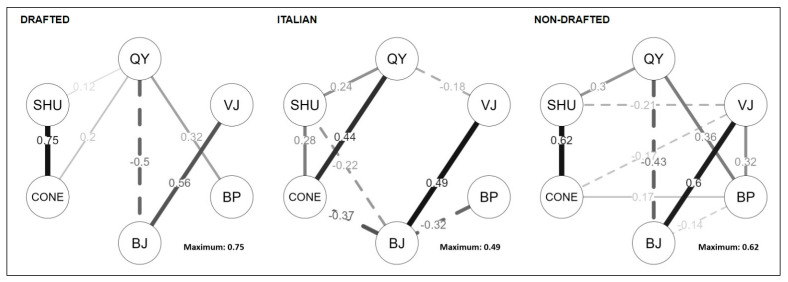
Network analysis of the performance profile of the three groups of players involved in the present study. Circular nodes represent the six Combine tests: QY = 40-yard dash, VJ = vertical jump, BP = bench press, BJ = broad jump, CONE = 3-cone drill, SHU = 20-yard shuttle. Solid lines represent positive relationships, while dashed lines represent negative ones. Highest relationships found were 0.75, 0.62, and 0.49 for drafted, non-drafted and Italian players, respectively.

Figure 1 uses network analysis to highlight performance differences and correlations among the three athlete groups. In Figure 1, the correlogram of all the six tests performed for each team is depicted. US players showed a very strong relationship between the 20-yard shuttle test and the L-cone agility test (r = 0.75 and r = 0.62, for drafted and non-drafted players, respectively), while in Italian players, this relationship was weaker (r = 0.28). A negative relationship was observed between the 40-yard sprint test and the broad jump in the US college players (r = −0.5 and r = −0.43 for drafted and non-drafted players, respectively) only. A significant relationship was detected between the 40-yard test and the maximum number of repetitions at the bench press test in US college players (r = 0.32 and r = 0.36 for drafted and non-drafted players, respectively) only. Italian players did not show significant correlations between the aforementioned tests. A positive relationship was found in both the US college group and the Division I Italian players, between vertical and broad jump tests (r = 0.56, r = 0.6, and r = 0.49 for drafted and non-drafted Italian players, respectively).

**Figure 2:**
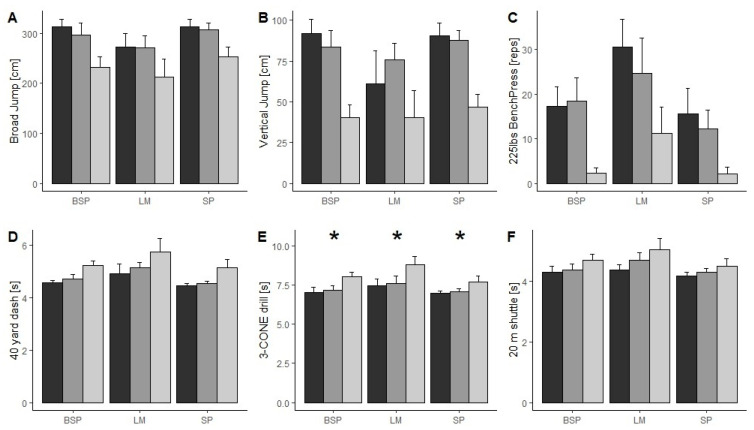
Mean and SD values for the six tests of the NFL Combine test battery performed by drafted US college players (black bars), non-drafted US college players (dark gray bars), and Italian Division I players (light gray bars); (Panel (**A**)) = 40-yard dash, (Panel (**B**)) = vertical jump, (Panel (**C**)) = bench press, (Panel (**D**)) = broad jump, (Panel (**E**)) = 3-cone drill, (Panel (**F**)) = 20-yard shuttle. * Denotes a significant team by player position interaction (*p* ≤ 0.05) between the Italian team and both the two US teams; BSP = big skills players; LM = lineman; SP = skills players.

Figure 2 presents the mean and SD values of the NFL Combine tests for the three examined groups of players and the three playing positions.

In all the six physical tests performed, Italian players obtained significantly lower results than both the drafted and the non-drafted US college players. Drafted US college players performed better than non-drafted players in all tests except for the 225 lbs bench press test for the BSP, where the latter, on average, performed more repetitions (17.3 ± 4.35 vs. 18.5 ± 5.15, for drafted and non-drafted players, respectively). Shapiro–Wilk tests showed that only broad jump scores were normally distributed (*p* > 0.001). All other tests in the NFL Combine battery have a Shapiro–Wilk *p*-value < 0.001, indicating non-normal distribution of the data. Therefore, results from broad jump test were analyzed with a parametric ANOVA while all other tests were analyzed using the equivalent nonparametric method of aligned rank transformation. In all tests, a significant main effect by player position and by team were observed (*p* < 0.001, d = 0.00–6.04). Moreover, in the 3-cone drill test, a significant team player/position interaction was observed (*p* = 0.0025, d = 0.22). Post hoc comparisons showed significative differences (*p* < 0.001, d > 2.00) between both drafted and non-drafted players compared to Italian players, in either BSP, LM and SP positions. For all the other NFL Combine tests there was no evidence of a significant interaction between players’ levels and position.

The comparisons between the players assessed in the present study and data collected in 2014 on Italian Division I players [15] revealed that, in 2020/2021, the average performance of players, grouped by position, was generally more elevated. The only performances that were not augmented were the vertical jump and broad jump for LM, and the number of repetitions at the bench press for BSP. Significant differences between teams were found for BSP in the 40-yard dash (*p* = 0.0230, t = 2.4447, d = 0.82), in the vertical jump (LM *p* = 0.0092, t = 2.8159, d = 0.94; SP *p* = 0.0139, t = 2.6455, d = 0.88; BSP = *p* = 0.0220, t = 2.5209, d = 0.84), in the 3-cone drill (LM *p* = 0.0448, t = 2.1081, d = 0.70; SP *p* = 0.0001, t = 4.5990, d = 1.53), and for LM in the number of repetitions in the bench press (*p* = 0.0262, t = 2.3842, d = 0.79).

## 4. Discussion

The aim of this study was to compare the results of the NFL Combine tests of three groups of athletes: Italian players, US college-drafted players and non-drafted players. Performances were normalized based on a player’s position, body mass, and stature. A secondary aim was to evaluate whether the performance of Italian Division I AF players has improved in recent years.

The network analysis showed strong relationships between the 20-yard shuttle test and the 3-cone drill test, between the 40-yard dash and broad jump test, and between the 40-yard dash and the bench press test in the two US player groups. These relationships allow us to better define the profile of Italian players in relation to college US players. Furthermore, strong correlations were observed between horizontal and vertical jump performances in all three teams, confirming the strong relationship between these two jump tests. Overall, network analysis shows that the performance and physical characteristics of US players were closer to the ideal AF Performance Model compared to the Italian players. Relationships between fitness parameters measured in US players suggest an explosive and more powerful profile compared to the Italian players in the study.

In contrast to the findings reported by Sierer and colleagues [14], the present study showed that BSP-drafted players showed lower results at the bench press test compared to the BSP non-drafted players. Moreover, Sierer and colleagues observed significant differences in the 40-yard dash and the 3-cone drill tests for all three positions, while only LM had better performance in the number of repetitions at the bench press test, and only SP in the vertical jump and 20-yard shuttle tests. This discrepancy between the present findings and those of Sierer et al. [14] may be attributed to the fact that they assessed professional NFL players, while the US players examined in the present study were college players. Moreover, by normalizing according to the criteria defined in this study, only a portion of the US college players were compared. Some of the differences found in the present study may be due to the inclusion/exclusion criteria adopted for the selection of the sample of players.

The comparison between the current Italian players and the data of the NFL Combine of Italian AF players competing in 2016, reported by Vitale et al. [15], revealed improved performances in the 40-yard dash, 3-cone drill test, and 20-yard shuttle tests across all playing positions. Conversely, better results in the broad jump test were observed only for SP and BSP, while improvements in the bench press test were limited to LM and SP, only. However, a decline in performance was detected for all playing positions in the vertical jump test. The countermovement jump test is an excellent parameter of explosive and reactive strength in advanced athletes, and an effective index of neuromuscular recovery following exercise [27]. An improved sprinting performance over a seven-year period, as shown for BSP, reflects an important milestone achieved by Italian players. Similarly, the enhanced performance on the bench press test observed in LM may reflect an increased emphasis on strength and conditioning among Italian AF players.

Consistently with the findings of Vitale and colleagues in 2016, the performance of Italian players was lower in all NFL Combine tests compared to US college players [15]. Even when considering college-level players rather than NFL professionals and normalizing the data by playing position, stature and weight, a substantial physical performance gap persists between Italian and US players.

## 5. Conclusions

The findings of this study, which employed the NFL Combine test battery, indicate that Italian American Football players consistently exhibit lower levels of physical fitness and performance compared to their US college counterparts. This discrepancy aligns with differences in technical proficiency between the two championships. Nevertheless, the popularity of AF has grown in Italy and across Europe, leading to notable improvement in players’ physical performance compared to previous years. For instance, current players showed better performances in the NFL Combine tests compared to Italian players competing in 2014. This improvement, observed over a seven-year period, suggests a more serious approach to strength and conditioning in young Italian players.

A key finding of this study is that even after normalizing the data for stature, weight, and playing position, significant performance gaps remain between Italian Division I players and US college players. While US players attain high levels of athleticism by the end of their college careers, many Italian players in Division I participate in the sport recreationally. This disparity represents a limitation in terms of achieving consistency for the present study, which unfortunately could not be overcome.

This and future studies on this topic could help address existing knowledge gaps. A further limitation of the present study is the absence of confidence intervals and effect size measures for edge weights in the estimated network. This limitation is a consequence of the software employed that lacks the requisite built-in functionality for calculating these measures. Another limitation is the relatively small sample size, involving only a single Italian AF team. While the sample size of the Italian AF players in this study may not fully represent the broader population, the normalization made by grouping players by position and playing level, paired with US players matched for stature and body weight, was designed to enhance comparability. Nevertheless, the findings should be interpreted with caution, as the representativeness of the sample cannot be guaranteed. Further research is necessary to explore the underlying reasons for the performance disparity between US and Italian players and to identify strategies for bridging this gap.

## 6. Practical Applications

The present findings provide valuable insights and reference data for coaches of Italian and European AF teams, helping them to understand and improve the physical performance of the players. Additionally, the network analysis offers a detailed understanding of the relationships between the physical tests included in the NFL Combine battery, which is widely used for fitness assessment across all playing levels. The rigorous inclusion criteria for the normalization of player performance outcomes in this study could serve as a useful tool for more precise evaluations. Although the results of this study may represent normative data for all European AF teams, given the limited research on non-American teams, it is important to consider that these findings are based on Italian AF players. The creation of a more extensive European database is highly recommended. Their generalizability to other European contexts may be constrained by variations in training methodologies, competition levels, and player characteristics. From a practical perspective, the results of this study can provide a starting point for evaluating performance and understanding the differences between the American and Italian AF performance models. Notably, the significant interactions observed in the 3-cone drill test suggest that this test is particularly effective for distinguishing player levels and positions. Thus, it may be preferable for talent identification and for assessing performance levels or the roles within AF players.

## Figures and Tables

**Table 1:** Combine test results of Italian players and of drafted and non-drafted college athletes are reported as mean and standard deviation; BSP = big skills players; LM = lineman; SP = skills players; * indicates a significant team by player position interaction (*p ≤* 0.05).

Performance Level	Player Position	40-Yard Dash [s]		Vertical Jump [cm]		Bench Press [reps]		Broad Jump [cm]		3-Cone Drills [s]		20-Yard Shuttle [s]	
**Italian**	***BSP***	5.21	(0.17)	40.3	(7.94)	2.20	(1.30)	232	(20.29)	8.03 *	(0.32)	4.69	(0.20)
	***LM***	5.80	(0.45)	36.4	(6.34)	11.0	(6.22)	205	(20.60)	8.80 *	(0.52)	5.05	(0.37)
	***SP***	5.13	(0.30)	46.5	(7.84)	2.14	(1.46)	253	(20.03)	7.72 *	(0.35)	4.50	(0.26)
**Drafted**	***BSP***	4.56	(0.09)	92.0	(8.66)	17.3	(4.35)	313	(14.61)	7.04 *	(0.31)	4.30	(0.21)
	***LM***	4.92	(0.27)	83.1	(7.66)	30.5	(7.26)	288	(16.54)	7.34 *	(0.29)	4.47	(0.19)
	***SP***	4.44	(0.09)	90.4	(8.06)	15.6	(5.74)	313	(15.51)	6.96 *	(0.17)	4.17	(0.12)
**Non-drafted**	***BSP***	4.69	(0.18)	83.3	(10.3)	18.5	(5.15)	296	(24.08)	7.19 *	(0.28)	4.39	(0.19)
	***LM***	5.14	(0.20)	75.8	(10.2)	24.7	(7.98)	271	(24.01)	7.59 *	(0.49)	4.70	(0.25)
	***SP***	4.53	(0.10)	87.5	(5.90)	12.3	(4.21)	307	(14.09)	7.07 *	(0.19)	4.29	(0.14)

## Data Availability

Data set available upon request from the authors.
